# Bis(2-amino-3-nitro­pyridinium) dichromate(VI)

**DOI:** 10.1107/S1600536808043018

**Published:** 2008-12-20

**Authors:** Samah Akriche, Mohamed Rzaigui

**Affiliations:** a Laboratoire de chimie des Matériaux, Faculté des Sciences de Bizerte, 7021 Zarzouna Bizerte, Tunisia

## Abstract

The title compound, (C_5_H_6_N_3_O_2_)_2_[Cr_2_O_7_], consists of 2-amino-3-nitro­pyridinium cations and discrete dichromate anions linked together by N—H⋯O and C—H⋯O hydrogen bonds, forming thick layers parallel to (101). Layer cohesion is ensured by N—H⋯O hydrogen bonding in addition to electrostatic and van der Waals inter­actions, forming a three-dimensional framework. The dichromate anion is located on a twofold axis that passes through its bridging O atom.

## Related literature

For related structures, see: Akriche & Rzaigui (2000 ▶); Khadhrani *et al.* (2006 ▶); Nicoud *et al.* (1997 ▶); Panunto *et al.* (1987 ▶); Sieroń (2007 ▶); Le Fur *et al.* (1998 ▶). For a discussion of hydrogen bonding, see: Desiraju (1989 ▶, 1995 ▶).
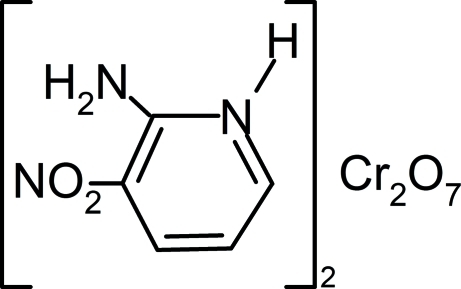


## Experimental

### 

#### Crystal data

(C_5_H_6_N_3_O_2_)_2_[Cr_2_O_7_]
*M* *_r_* = 496.26
Monoclinic, 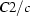

*a* = 14.799 (2) Å
*b* = 7.464 (3) Å
*c* = 17.870 (5) Å
β = 116.71 (4)°
*V* = 1763.3 (11) Å^3^
*Z* = 4
Mo *K*α radiation
μ = 1.31 mm^−1^
*T* = 298 K
0.25 × 0.23 × 0.19 mm

#### Data collection

Enraf–Nonius TurboCAD-4 diffractometer
Absorption correction: none
3444 measured reflections
2123 independent reflections
1562 reflections with *I* > 2σ(*I*)
*R*_int_ = 0.021
2 standard reflections frequency: 120 min intensity decay: 3%

#### Refinement

*R*[*F* ^2^ > 2σ(*F* ^2^)] = 0.039
*wR*(*F* ^2^) = 0.106
*S* = 1.04
2123 reflections
132 parameters
H-atom parameters constrained
Δρ_max_ = 0.44 e Å^−3^
Δρ_min_ = −0.37 e Å^−3^

### 

Data collection: *CAD-4 EXPRESS* (Enraf–Nonius, 1994 ▶); cell refinement: *CAD-4 EXPRESS*; data reduction: *XCAD4* (Harms & Wocadlo, 1996 ▶); program(s) used to solve structure: *SHELXS97* (Sheldrick, 2008 ▶); program(s) used to refine structure: *SHELXL97* (Sheldrick, 2008 ▶); molecular graphics: *ORTEP-32 for Windows* (Farrugia, 1998 ▶); *DIAMOND* (Brandenburg & Putz, 2005 ▶); software used to prepare material for publication: *WinGX* publication routines (Farrugia, 1999 ▶).

## Supplementary Material

Crystal structure: contains datablocks I, global. DOI: 10.1107/S1600536808043018/dn2417sup1.cif

Structure factors: contains datablocks I. DOI: 10.1107/S1600536808043018/dn2417Isup2.hkl

Additional supplementary materials:  crystallographic information; 3D view; checkCIF report

## Figures and Tables

**Table 1:** Hydrogen-bond geometry (Å, °)

*D*—H⋯*A*	*D*—H	H⋯*A*	*D*⋯*A*	*D*—H⋯*A*
N1—H1⋯O2	0.86	1.87	2.707 (3)	165
N2—H2*A*⋯O4	0.86	2.17	2.974 (4)	155
N2—H2*B*⋯O6	0.86	2.06	2.654 (4)	125
N2—H2*B*⋯O6^i^	0.86	2.59	3.061 (4)	116
C3—H3⋯O4^ii^	0.93	2.58	3.494 (4)	167
C4—H4⋯O3^iii^	0.93	2.50	3.337 (4)	150
C5—H5⋯O2^iv^	0.93	2.34	3.232 (4)	160

Symmetry codes: (i) 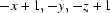; (ii) 
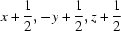
; (iii) 
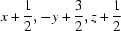
; (iv) 
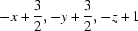
.

**Fig. 1.**
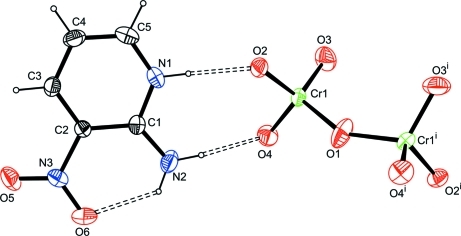
An ORTEP view of (I) with the atom-labelling scheme. Displacement ellipsoids are drawn at the 30% probability level. H atoms are represented as small spheres of arbitrary radii. Hydrogen bonds are represented as dashed lines. [Symmetry code: (i) -x+1, y, -z+1/2]

**Fig. 2.**
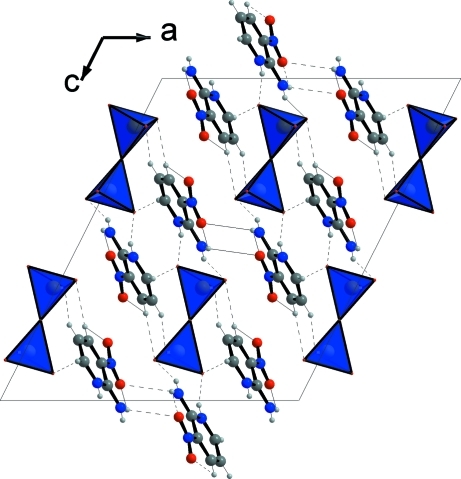
Projection of (I) along the b axis.

## supplementary crystallographic information

### Comment

A new engineering strategy using organic-inorganic hybrid materials have appeared over the past years. The challenge was to combine the advantages of organic crystals and those of the inorganic materials. As a part of our study of crystal packing in amino-nitro "push-pull" system, a new organic-inorganic salt, bis (2-amino-3-nitropyridinium) dichromate (I) have been synthesized.

The dichromate anion has a binary internal symmetry since its bridging oxygen atom is located on a twofold axis, and so is built by only one independent (CrO_4_) group. This later with one independent (2-NH_2_-3-NO_2_C_5_H_3_NH)_+_ cation constitute the asymmetric unit of (I) (Fig. 1).

As expected, the main geometrical features of anion agree with those previously observed for this group in other coumpounds (Sieroń, 2007; Khadhrani *et al.*,2006). The bond lengths and the angles within the cation are comparable with those observed for 2-amino-3-nitropyridinium dihydrogenphosphate (Akriche *et al.*,2000), 2-amino-3-nitropyridinium hydrogensulfate(Le Fur *et al.*, 1998) and 2-amino-3-nitropyridinium chloride (Nicoud *et al.*,1997).

The dichromate and organic entities manifest different interactions (electrostatic, H-bonds, Van Der Waals) to keep up the three-dimensionel network cohesion (Fig. 2). The main links are from the N—H···O bonds (Table 1) with H···O bond lengths falling in the range from 1.87–2.59 Å.

Long C—H···O contacts occur between cations and cation-anion moities with C···O bond lengths ranging from 3.494 (4)–3.232 (4)Å (Desiraju, 1989; Desiraju, 1995).

It's worth noticing the intracation contact N2—H2B···O6 (see Table 1 for symmetry code) which is always present in nitroaniline in which nitro and amino groups are *ortho* to one another, as clearly shown in a study of hydrogen patterns of nitroaniline derivatives (Panunto *et al.*, 1987). This situation precludes the rotation of the nitro group with respect to pyridinium ring. The angle between the planes of the NO_2 _group and the heterocycle is 7.98° for cation, indicating a coplanar geometry.

### Experimental

0.004 mol of 2-amino-3-nitropyridine was dissolved in 20 ml of pure acetic acid. 5 ml solution containing 0.004 mol of CrO_3_ was added drop by drop under stirring at 333 K. The obtained solution is slowly evaporated at the ambiant temperature. After some days, Brown single crystals of the title compound are formed in the reactionnel midle.

### Crystal data

**Table:**

(C_5_H_6_N_3_O_2_)_2_[Cr_2_O_7_]	*F*(000) = 1000
*M**_r_* = 496.26	*D*_x_ = 1.869 Mg m^−^^3^
Monoclinic, *C*2/*c*	Mo *K*α radiation, λ = 0.71073 Å
Hall symbol: -C 2yc	Cell parameters from 25 reflections
*a* = 14.799 (2) Å	θ = 9–11°
*b* = 7.464 (3) Å	µ = 1.31 mm^−^^1^
*c* = 17.870 (5) Å	*T* = 298 K
β = 116.71 (4)°	Diamond-shaped, brown
*V* = 1763.3 (11) Å^3^	0.25 × 0.23 × 0.19 mm
*Z* = 4	

### Data collection

**Table:**

Enraf–Nonius TurboCAD-4 diffractometer	*R*_int_ = 0.021
Radiation source: fine-focus sealed tube	θ_max_ = 28.0°, θ_min_ = 2.6°
graphite	*h* = −19→19
non–profiled ω scans	*k* = 0→9
3444 measured reflections	*l* = −10→23
2123 independent reflections	2 standard reflections every 120 min
1562 reflections with *I* > 2σ(*I*)	intensity decay: 3%

### Refinement

**Table:**

Refinement on *F*^2^	Primary atom site location: structure-invariant direct methods
Least-squares matrix: full	Secondary atom site location: difference Fourier map
*R*[*F*^2^ > 2σ(*F*^2^)] = 0.039	Hydrogen site location: inferred from neighbouring sites
*wR*(*F*^2^) = 0.106	H-atom parameters constrained
*S* = 1.04	*w* = 1/[σ^2^(*F*_o_^2^) + (0.0546*P*)^2^ + 0.8978*P*] where *P* = (*F*_o_^2^ + 2*F*_c_^2^)/3
2123 reflections	(Δ/σ)_max_ = 0.002
132 parameters	Δρ_max_ = 0.44 e Å^−^^3^
0 restraints	Δρ_min_ = −0.37 e Å^−^^3^

### Special details

**Table:**

Geometry. All esds (except the esd in the dihedral angle between two l.s. planes) are estimated using the full covariance matrix. The cell esds are taken into account individually in the estimation of esds in distances, angles and torsion angles; correlations between esds in cell parameters are only used when they are defined by crystal symmetry. An approximate (isotropic) treatment of cell esds is used for estimating esds involving l.s. planes.
Refinement. Refinement of *F*^2^ against ALL reflections. The weighted *R*-factor *wR* and goodness of fit *S* are based on *F*^2^, conventional *R*-factors *R* are based on *F*, with *F* set to zero for negative *F*^2^. The threshold expression of *F*^2^ > σ(*F*^2^) is used only for calculating *R*-factors(gt) *etc*. and is not relevant to the choice of reflections for refinement. *R*-factors based on *F*^2^ are statistically about twice as large as those based on *F*, and *R*- factors based on ALL data will be even larger.

### Fractional atomic coordinates and isotropic or equivalent isotropic displacement parameters (Å^2^)

**Table:**

	*x*	*y*	*z*	*U*_iso_*/*U*_eq_	
Cr1	0.51253 (3)	0.65455 (6)	0.34930 (3)	0.03488 (15)	
O1	0.5000	0.5931 (6)	0.2500	0.0760 (11)	
O2	0.63121 (14)	0.6372 (3)	0.41598 (13)	0.0434 (5)	
O3	0.4736 (2)	0.8522 (3)	0.35084 (18)	0.0672 (7)	
O4	0.44934 (17)	0.5121 (3)	0.37400 (14)	0.0544 (6)	
O5	0.7372 (2)	−0.1246 (3)	0.67939 (16)	0.0607 (7)	
O6	0.6258 (2)	−0.0987 (3)	0.55077 (17)	0.0687 (7)	
N1	0.70398 (18)	0.4291 (3)	0.55486 (16)	0.0416 (6)	
H1	0.6730	0.5020	0.5139	0.050*	
N2	0.58479 (19)	0.2213 (4)	0.47828 (16)	0.0544 (7)	
H2A	0.5568	0.3004	0.4397	0.065*	
H2B	0.5594	0.1155	0.4719	0.065*	
N3	0.69260 (19)	−0.0377 (3)	0.61537 (17)	0.0425 (6)	
C1	0.6667 (2)	0.2622 (4)	0.54739 (17)	0.0351 (6)	
C2	0.72292 (19)	0.1482 (3)	0.61610 (16)	0.0317 (5)	
C3	0.8061 (2)	0.2088 (4)	0.68474 (18)	0.0402 (6)	
H3	0.8409	0.1320	0.7296	0.048*	
C4	0.8388 (2)	0.3827 (4)	0.6881 (2)	0.0499 (8)	
H4	0.8953	0.4252	0.7345	0.060*	
C5	0.7856 (2)	0.4899 (4)	0.6213 (2)	0.0494 (8)	
H5	0.8063	0.6077	0.6218	0.059*	

### Atomic displacement parameters (Å^2^)

**Table:**

	*U*^11^	*U*^22^	*U*^33^	*U*^12^	*U*^13^	*U*^23^
Cr1	0.0320 (2)	0.0429 (3)	0.0274 (2)	0.0004 (2)	0.01125 (17)	0.00519 (19)
O1	0.073 (2)	0.122 (3)	0.0321 (17)	0.000	0.0232 (17)	0.000
O2	0.0357 (9)	0.0461 (11)	0.0406 (11)	−0.0011 (9)	0.0101 (9)	0.0065 (9)
O3	0.0642 (15)	0.0518 (14)	0.0762 (18)	0.0208 (12)	0.0233 (14)	0.0154 (12)
O4	0.0483 (12)	0.0604 (14)	0.0583 (13)	−0.0093 (11)	0.0272 (11)	0.0052 (11)
O5	0.0850 (18)	0.0428 (13)	0.0624 (15)	0.0050 (12)	0.0402 (15)	0.0172 (11)
O6	0.0712 (16)	0.0492 (13)	0.0703 (17)	−0.0253 (12)	0.0182 (14)	−0.0142 (12)
N1	0.0445 (13)	0.0357 (12)	0.0502 (15)	0.0080 (11)	0.0263 (12)	0.0123 (11)
N2	0.0428 (14)	0.0691 (18)	0.0389 (14)	−0.0034 (13)	0.0072 (12)	0.0105 (13)
N3	0.0505 (14)	0.0339 (12)	0.0511 (15)	−0.0035 (11)	0.0298 (12)	−0.0016 (12)
C1	0.0334 (12)	0.0418 (15)	0.0339 (14)	0.0036 (12)	0.0186 (11)	0.0044 (12)
C2	0.0343 (12)	0.0304 (12)	0.0323 (13)	0.0013 (11)	0.0168 (11)	0.0004 (11)
C3	0.0407 (14)	0.0429 (15)	0.0327 (14)	0.0044 (12)	0.0128 (12)	0.0036 (12)
C4	0.0453 (16)	0.0486 (18)	0.0464 (17)	−0.0110 (14)	0.0122 (14)	−0.0121 (14)
C5	0.0550 (18)	0.0321 (15)	0.068 (2)	−0.0080 (13)	0.0342 (17)	−0.0074 (14)

### Geometric parameters (Å, °)

**Table:**

Cr1—O3	1.588 (2)	N2—H2A	0.8600
Cr1—O4	1.603 (2)	N2—H2B	0.8600
Cr1—O2	1.625 (2)	N3—C2	1.457 (3)
Cr1—O1	1.7601 (14)	C1—C2	1.416 (4)
O1—Cr1^i^	1.7601 (14)	C2—C3	1.366 (4)
O5—N3	1.219 (3)	C3—C4	1.377 (4)
O6—N3	1.221 (4)	C3—H3	0.9300
N1—C5	1.336 (4)	C4—C5	1.356 (5)
N1—C1	1.344 (4)	C4—H4	0.9300
N1—H1	0.8600	C5—H5	0.9300
N2—C1	1.320 (4)		
			
O3—Cr1—O4	110.50 (14)	N2—C1—N1	118.1 (3)
O3—Cr1—O2	110.01 (13)	N2—C1—C2	127.3 (3)
O4—Cr1—O2	108.49 (11)	N1—C1—C2	114.6 (2)
O3—Cr1—O1	112.62 (17)	C3—C2—C1	121.3 (3)
O4—Cr1—O1	107.14 (15)	C3—C2—N3	118.2 (2)
O2—Cr1—O1	107.93 (9)	C1—C2—N3	120.4 (2)
Cr1^i^—O1—Cr1	149.8 (3)	C2—C3—C4	120.5 (3)
C5—N1—C1	124.7 (3)	C2—C3—H3	119.7
C5—N1—H1	117.7	C4—C3—H3	119.7
C1—N1—H1	117.7	C5—C4—C3	117.7 (3)
C1—N2—H2A	120.0	C5—C4—H4	121.2
C1—N2—H2B	120.0	C3—C4—H4	121.2
H2A—N2—H2B	120.0	N1—C5—C4	121.1 (3)
O5—N3—O6	123.8 (3)	N1—C5—H5	119.4
O5—N3—C2	117.6 (3)	C4—C5—H5	119.4
O6—N3—C2	118.6 (3)		

Symmetry codes: (i) −*x*+1, *y*, −*z*+1/2.

### Hydrogen-bond geometry (Å, °)

**Table:**

*D*—H···*A*	*D*—H	H···*A*	*D*···*A*	*D*—H···*A*
N1—H1···O2	0.86	1.87	2.707 (3)	165
N2—H2A···O4	0.86	2.17	2.974 (4)	155
N2—H2B···O6	0.86	2.06	2.654 (4)	125
N2—H2B···O6^ii^	0.86	2.59	3.061 (4)	116
C3—H3···O4^iii^	0.93	2.58	3.494 (4)	167
C4—H4···O3^iv^	0.93	2.50	3.337 (4)	150
C5—H5···O2^v^	0.93	2.34	3.232 (4)	160

Symmetry codes: (ii) −*x*+1, −*y*, −*z*+1; (iii) *x*+1/2, −*y*+1/2, *z*+1/2; (iv) *x*+1/2, −*y*+3/2, *z*+1/2; (v) −*x*+3/2, −*y*+3/2, −*z*+1.
